# Category-specific neuronal activity in left and right auditory cortex and in medial geniculate body of monkeys

**DOI:** 10.1371/journal.pone.0186556

**Published:** 2017-10-26

**Authors:** Elena Selezneva, Elena Oshurkova, Henning Scheich, Michael Brosch

**Affiliations:** 1 Specal Lab Primate Neurobiology, Leibniz-Institute for Neurobiology, Magdeburg, Germany; 2 Department Auditory Learning and Speech, Leibniz-Institute for Neurobiology, Magdeburg, Germany; Universidad de Salamanca, SPAIN

## Abstract

We address the question of whether the auditory cortex of the left and right hemisphere and the auditory thalamus are differently involved in the performance of cognitive tasks. To understand these differences on the level of single neurons we compared neuronal firing in the primary and posterior auditory cortex of the two hemispheres and in the medial geniculate body in monkeys while subjects categorized pitch relationships in tone sequences. In contrast to earlier findings in imaging studies performed on humans, we found little difference between the three brain regions in terms of the category-specificity of their neuronal responses, of tonic firing related to task components, and of decision-related firing. The differences between the results in humans and monkeys may result from the type of neuronal activity considered and how it was analyzed, from the auditory cortical fields studied, or from fundamental differences between these species.

## Introduction

In most speech signals and music, information is contained in the pitch relationships between adjacent sound elements. Sentence type can easily be recognized on the basis of pitch contour alone [1]. Melody recognition relies on the sequential up-and-down patterning; transpositions that preserve this patterning are often recognized as the same melody [2]. The identification of pitch relationships, or the direction of tone steps, is independent of the absolute frequency of the tones and of the size of the tone step, i. e., it is categorical. These perceptual abilities are particularly well developed in humans, although some nonhuman animals, including monkeys ([3], [4], [5], [6], [7]), ferrets ([8], [9]), dolphins ([10]), and starlings ([11]) are also able to identify pitch relationships between sounds. It is thus possible that humans and nonhuman animals share brain mechanisms for obtaining pitch relationships. Animal studies can be used to identify these possible common mechanisms on the level of single neurons or small neuronal groups.

Previously we described neuronal firing in the primary and caudomedial auditory cortex of monkeys while they categorized tone steps ([12], [13]). Monkeys had to grasp and hold a bar to trigger a sequence of tones of variable frequencies, and had to release the lever after the frequency has stepped down. We found two types of neuronal responses (phasic and tonic) that were differently related to this categorization task.

Phasic responses had short latencies and durations in the range of tens of milliseconds, and their magnitude was related to the three classes of tone steps: downsteps (from a higher to lower frequency), upsteps (from lower to higher) and repeats of the same frequency. When we analyzed the development of the phasic responses during the sequence we found that the response to a given tone was increased relative to that of the preceding tone for downsteps but not for upsteps and frequency repeats; thus the response increase reflected the two behavioral meanings of the tone steps, namely bar release and bar holding.

Tonic responses were temporally related to coincident with specific phases of the task. Of particular relevance were neurons with a tonic increase of their firing in more posterior parts of the auditory cortex which were related to and predicted the behavioral responses of the monkeys. No such relationship was found in the phasic responses.

These two types of neuronal responses were observed in the left auditory cortex ([12], [13]). Neuropsychological studies in humans, however, have indicated that the right auditory cortex might be more relevant for categorizing tone steps ([14], [15]). This observation is in line with noninvasive imaging studies in healthy subjects. Brechmann and Scheich [16] found stronger activation in right than in left auditory cortex when subjects were engaged in categorizing the direction of frequency sweeps. Foxton and colleagues [17] showed that humans with poor performance in such categorization tasks had weaker activation in the right auditory cortex relative to the left auditory cortex.

Motivated by these reports, we wanted to follow four lines of investigation. (1) Are different neuronal mechanisms for categorizing tone steps implemented in the left and right auditory cortex. To address this question we studied corresponding fields in the right auditory cortex of the same monkeys in which we had previously studied the left auditory cortex (([12], [13]). (2) To further clarify whether the response preference for downsteps is a result of learning the categorization task or simply represented a sampling bias towards neurons whose best frequency corresponded better to the frequency composition of tone steps, we studied the auditory cortex of a control monkey who was only passively exposed to the same tone sequences and was not able to perform the categorization task. (3) There are conflicting results whether activity in the auditory cortex is also related to behavioral responses during other auditory categorization ([18], [19]) or discrimination tasks ([20], [21], [22], [23]). To allow a better comparison of our results with these other studies we performed a choice probability analysis ([24]). (4) Finally, we wondered whether the neuronal activity related to the categorization task is generated in auditory cortex or is also present subcortically. We therefore also studied the medial geniculate body of the same monkeys during task performance.

## Materials & methods

The experimental approach and the methods of the present study are similar to those described in our previous studies ([5], [12], [13], [25]).

### Subjects

Data of the present report are from three adult male cynomolgus monkeys (macaca fascicularis). All of them were born and housed in the breeding colony of the Leibniz-Institute for Neurobiology, Magdeburg prior the study. Two of the monkeys had previously participated in the same categorization study involving left auditory cortex (monkey B and monkey F). The third monkey (monkey C) was naive to the categorization task used here and had participated in other physiological and behavioral studies ([26]), in which he was presented with an audiovisual sequence and had to report the cessation of the visual stimuli. A headholder (‘halo’) device was implanted under deep anesthesia (ketamine HCl [4 mg/kg] and xylazine [5 mg/kg]) onto the monkey's head to allow atraumatic head restraint. It consisted of three strong arches that closely encircle the occipital, supra-orbital and mid-sagittal ridges of the head. This helmet-like piece was firmly and permanently attached to the head by means of several counteracting stainless steel bolts with sharpened points, which were advanced by rotation through the intact skin and soft tissue until they lodge firmly against the skull. Subsequently, monkeys received one or two recording chamber implants, positioned in the left (all monkeys) or in the right (monkeys F and B) temporal regions of the skull, for micro-electrode recordings from the auditory cortex and the medial geniculate body (MGB). For the implantation, a piece of bone was removed with a trephine (diameter: 21 mm) and an externally threaded stainless steel cylinder was screwed into the slightly undersized hole. All surgical operations on the animals were performed under deep general anesthesia and were followed by a full course of antibiotic (Baytril [0,2 ml/kg]) and analgesic (Carprofen [0,1 ml/kg]) treatment during which they were monitored several times per day as long as required. Throughout the experiments, the monkeys were housed in groups in cages with environmental enrichments in the form of toys for play inside their cages and audio visual entertainment placed outside the cages. To motivate behavior during the experiment, the monkeys had restricted access to water for 20 h before behavioral sessions. At least one day per week, they were allowed to access water ad libitum. Dry food and fruits were always available ad libitum in the home cage. The monkeys were euthanized by an overdose of Nembutal (0,2 g/kg) at the conclusion (monkey F) or seven months after the end of the current recordings (monkey B and monkey C). The study was approved by the authority for animal care and ethics (Tierversuchs-Ethik-Kommission) of the federal state of Saxony Anhalt (No. 43.2-42502/2-253 IfN) and conformed with the rules for animal experimentation of the European Communities Council Directive (86/609/EEC).

### Behavioral procedure

**Fig 1:**
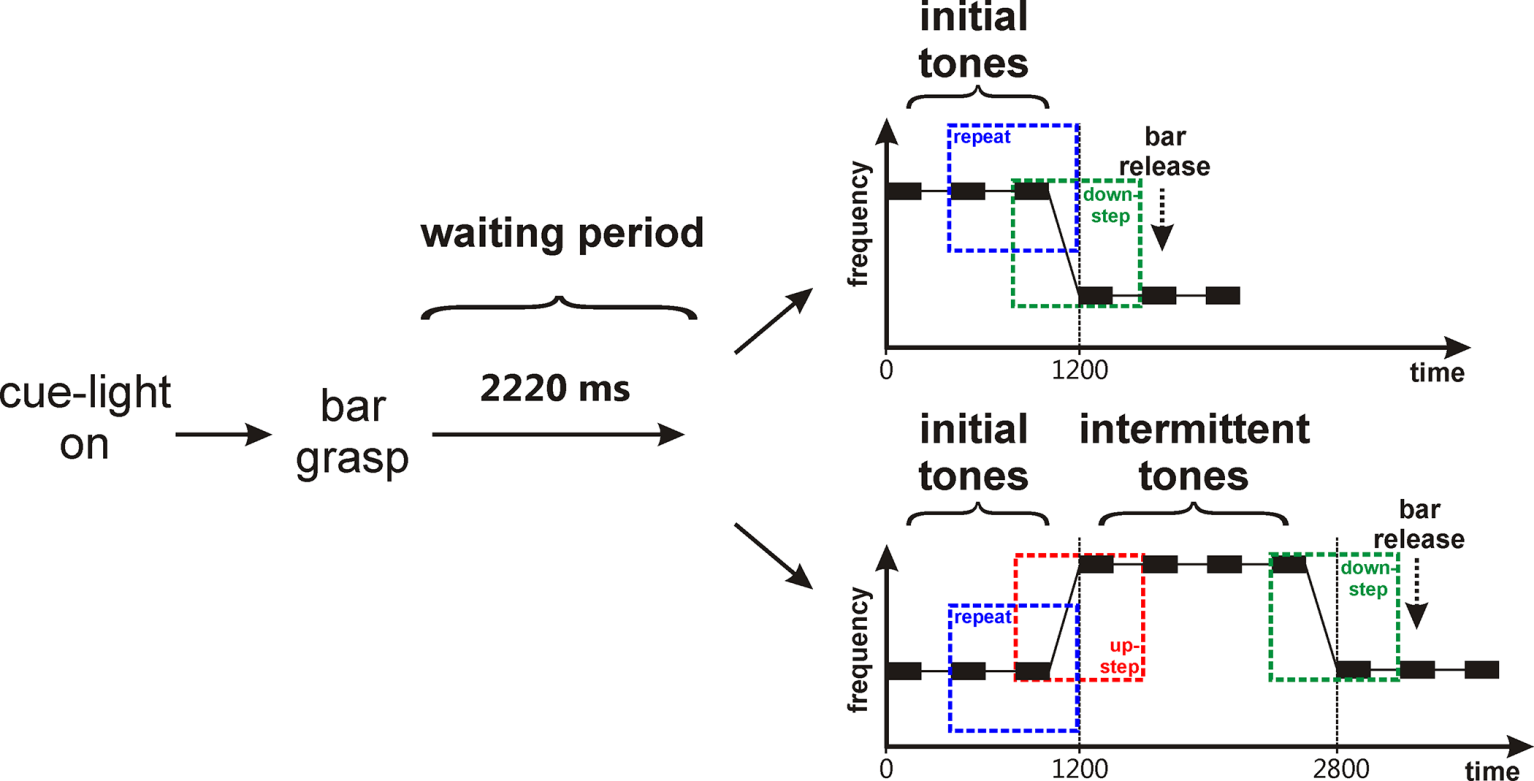
Auditory categorization task. A trial started by turning on a cue light, which was the signal for the monkeys to grasp a touch bar. After a waiting period of 2220 ms, a sequence of up to ten tones started (black rectangles). Two types of tone sequences were used. The first type was composed of three initial tones with the same frequency followed by up to three tones of lower frequency. It consisted of one downstep (green square) and several frequency repeats of which the one marked by the blue square was taken to analyze category sensitivity of neurons. The second type was composed of three initial tones, followed by four intermittent tones of higher frequency, which were followed by up to three tones of lower frequency. This sequence thus also consisted of one upstep (red square) in addition to the downstep and the frequency repeats. The monkeys had to release the bar after the occurrence of a downstep (downward pointing arrow).

Two monkeys (monkeys F and B) were trained to categorize the direction of tone steps (Fig 1). At the beginning of each trial a cue light was turned on, in response to which the monkeys had to grasp a touch bar within the following 3 s. After a waiting period of 2220 ms of continued bar holding, a sequence of up to ten tones started. Each of the tones was presented at a sound pressure level of ~ 60 dB SPL, had a duration of 200 ms and was followed by a silent intertone interval of 200 ms. The three initial tones had the same frequency which, for each trial, was randomly drawn from a set of ten frequencies, separated by 0.5 octaves. The lowest frequency varied from 300 to 600 Hz between experimental sessions but was fixed during individual sessions. The three initial tones were followed by up to three tones of the same lower frequency, either directly, or after the presentation of four (in some sessions three to five) intermittent tones at a higher frequency. Thus sequences consisted of up to seven frequency repeats, one downstep and no or one upstep. The size of the downsteps and upsteps was either small (0.5 octaves) or large (1 octave). The monkeys' task was to signal, by bar release, the occurrence of a downstep. If they did so 240 to 1240 ms after the onset of the first tone with a lower frequency (i. e., after the second tone of a downstep), the tone sequence and the cue light were turned off immediately and a water reward was administered. Bar releases at all other times were not rewarded and resulted in a 5-s timeout from the experiment. The control monkey (monkey C) was only passively exposed to the same tone sequences.

### Electrophysiological recordings

We used a multielectrode drive (System Eckhorn, Thomas Recording; impedance between 2 and 2.5 MΩ measured at 1 kHz) to record the action potentials fired by a small group of neurons in the vicinity of each electrode (multiunit activity). For the recordings from the primary and the caudomedial auditory cortex of the right hemisphere (RAC) we used the same approach as in our previous study in which we recorded from the same two auditory fields in the left hemisphere (LAC; [12]). The two fields were identified physiologically by the spatial distributions of best frequencies that were characteristic for primary and caudomedial auditory cortex ([27]). To assess the best frequency and the frequency response area of a recording site we presented a random sequence of 400 pure tones at 40 different frequencies (see [25] for further details). In the present report we included only sites with excitatory responses to sounds. We refrained from isolating the action potentials of single neurons from our multiunit recordings because as shown in our previous studies (1) the small number of single neurons precluded statistical verification of the results of the category-sensitivity in LAC ([12]) and (2) only a small fraction of single neurons exhibited tonic responses ([13]). Previously we showed that single units in LAC can also exhibit ramping activity ([13]) and firing that is related to the tone steps category ([12]) and to non-auditory events ([25], [28]).

For the recordings from the right medial geniculate body (MGB), we advanced 3 to 7 electrodes through the same chamber that was used for the cortical recordings. Electrode tracks were oriented at an angle of ~ 45 degree in the dorsolateral plane such that the electrodes penetrated the dura approximately at a right angle, although more laterally than for the recordings from primary and caudomedial auditory cortex. Subsequently the electrodes were advanced by at least 15 mm before we searched for neuronal responses to pure tones of various frequencies. Such responses were found and recorded at depths between 17 and 24 mm below the brain surface, in good correspondence with the location of the MGB estimated from the brain atlas ([29]).

**Fig 2:**
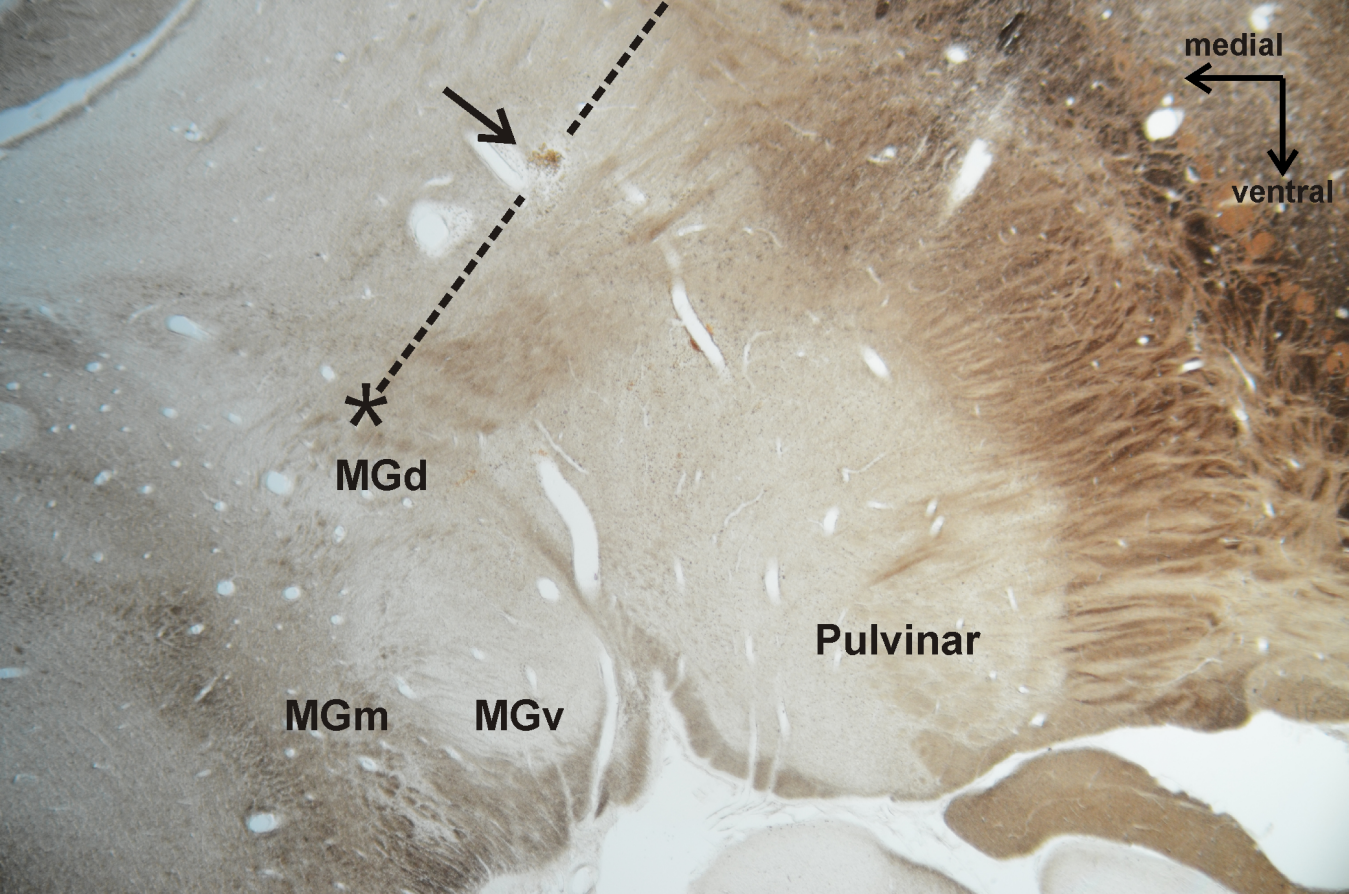
Frontal section through the medial geniculate body (MGB) and adjacent thalamic areas of monkey B (differential interference contrast for myeloarchitecture). The dashed line indicates the electrode track. The arrow shows the iron staining visualized by the Prussian blue reaction. The star shows the location of one recording site three millimeters more medially and ventrally. MGv: ventral division of the MGB; MGd: dorsal division of the MGB; MGm: medial division of the MGB.

For one recording site, we verified that the electrode was successfully placed in the dorsal division of the MGB (MGd, Fig 2). Neurons at this site responded to low frequency tones between 80 and 600 Hz with a latency of ~15 ms. Subsequently the electrode was retracted by 3 mm dorsolaterally from the recording site to a site where a small iron deposit was made by passing a train of biphasic current pulses with an amplitude of 20 μA at 1 kHz over a period of 10 s. Five months later, the monkey was given an overdose of Nembutal and was perfused transcardially with saline followed by 4% paraformaldehyde. The iron deposit was visualized by the Prussian blue reaction on frontal sections in which the myeloarchitecture was visualized by differential interference contrast imaging. Although no histological reconstructions were available for other recording sites in this and the other monkey, many of the sites were likely located in the ventral division of the MGB (MGv). This is suggested by finding that the best frequencies remained relatively constant when we moved the electrode to sites more ventrally or more dorsally from a site which was analyzed in the current study, as established in new world monkeys ([30]).

### Data analyses

#### Phasic responses

**Fig 3:**
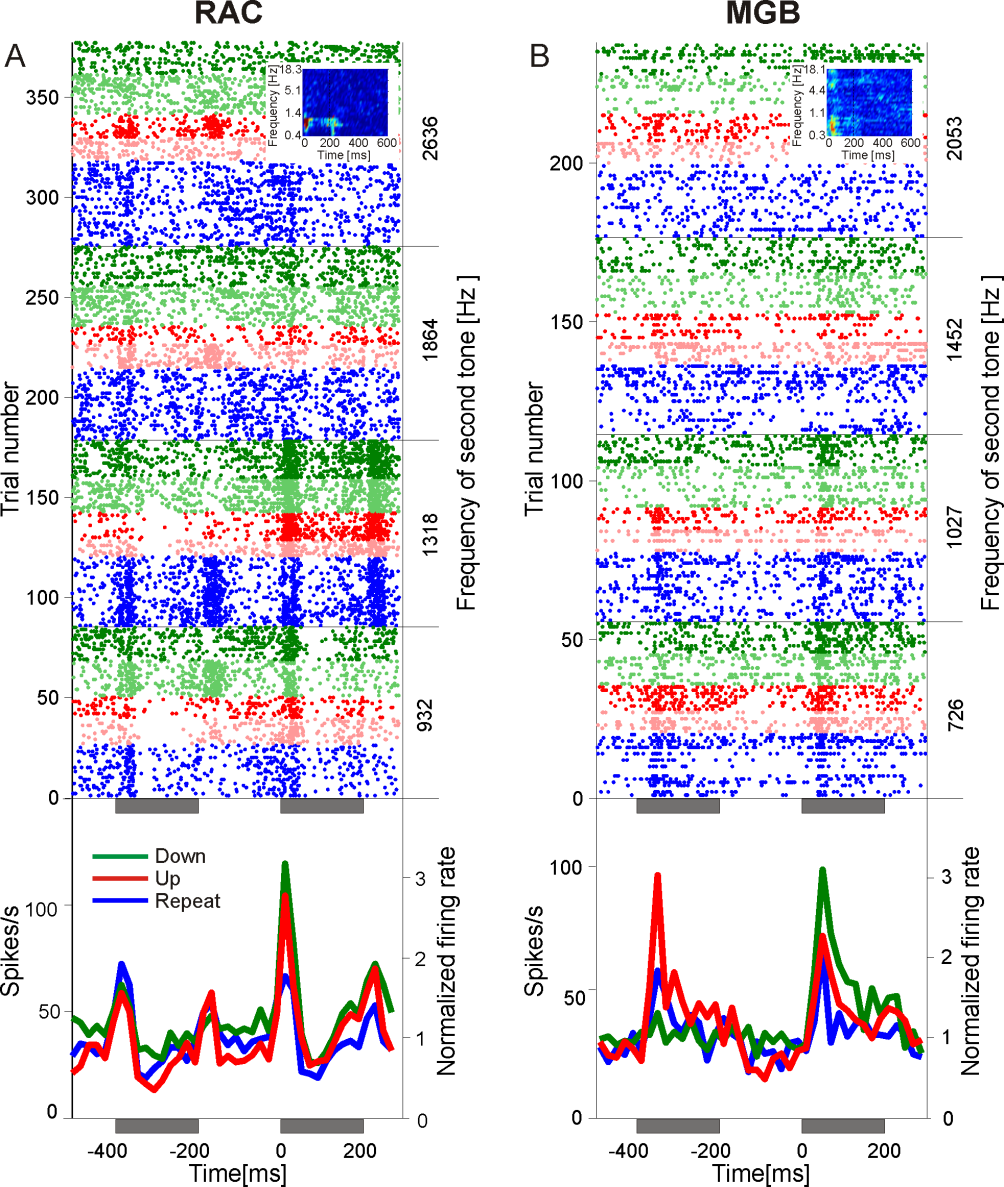
Phasic responses to three classes of tone steps in the auditory forebrain. (A) Multiunit activity recorded in right auditory cortex (RAC). In the raster display, the dots in each horizontal raster line indicate when the multiunit fired spikes relative to the 2nd tone of different exemplars of downsteps (green), upsteps (red), and frequency repeats (blue). Small tone steps (0.5 octaves) are depicted by pale dots, large tone steps (1 octave) by bold dots. Gray bars underneath the abscissas indicate the 1st and the 2nd tone of the tone step. The left ordinate indicates the trial number. The right ordinate indicates the frequency of the 2nd tone of the tone steps, i. e., the trigger tone. The post stimulus time histograms underneath the rastergrams show the time courses of the normalized spike rate averaged across all downsteps, all upsteps, and all frequency repeats. The inset shows the frequency response area of this multiunit. (B) Multiunit activity recorded in medial geniculate body (MGB).

To analyze phasic responses of individual multiunits to the three classes of tone steps we computed three post stimulus time histograms (PSTHs) from the neuronal firing with a bin size of 20 ms and a shift window of 1 ms, each of which was triggered on the onset of tones at selected positions in the sequence. For upsteps, the trigger tone was the 4^**th**^ position; for downsteps, this was either the 4^**th**^ position or the 8^**th**^ position; for frequency repeats, this was the 3^**rd**^ position. We used only those tone steps in which the trigger tone was from the same range of 1.5 octaves, starting from 1 octave above the lowest frequency tested during an experimental session. This resulted in the use of 4 exemplars of frequency repeats and, because there were small and large tone steps, 8 exemplars of downsteps and 8 exemplars of upsteps. Typical testing ranges are shown in Fig 3. The remaining tone steps ending on tones outside the 1.5-octave range were needed for the behavioral testing to make sure that the monkeys were indeed categorizing the tone sequences and not using other features of the tone sequences for correct performance. They were not used for the assessment of the phasic responses because for a given trigger tone frequency no complete sets of tone steps could be compiled which consisted of one frequency repeat, of small and large upsteps and the two corresponding downsteps.

To ease comparisons across multiunits, the firing rate was normalized by dividing it by the firing rate in the 200-ms period before sequence onset. The bin with the maximal firing in the initial 60 ms of the PSTH was taken as the magnitude of a multiunit's response to a tone step and termed 'phasic response strength'. This bin was found at a latency of 30.6 (± 7.2) ms in RAC, 21.9 (± 8.4) ms in MGB and 27.1 (± 7.5) ms in LAC. In addition, we analyzed the temporal development of the responses to the consecutive tones in the sequence. For this purpose, we calculated the 'phasic response increase', which was the difference between the phasic response strength to the trigger tone and the phasic response strength to the preceding tone, divided by the phasic response strength to the preceding tone. Phasic response increases were expressed as percentages. Positive values indicated that the response increased from the preceding to the trigger tone, negative values indicated response decreases.

In this report, only a subset of all recorded multiunits could be used for the analysis of the phasic responses to the three classes of tone steps. The number of multiunits was significantly reduced because we required that (1) each of the exemplars of tone steps was presented at least three times in correctly performed trials (this criterion was necessary to diminish the influence of outliers) and that (2) the phasic response strength of at least one of the exemplars of tone steps was significantly greater than 1 (t-test, p < 0.05; most were < 0.01). This guaranteed that the analysis included only multiunits that were tested with tones inside the frequency response area, of which many were tested with at least one trigger tone ≤ 0.5 octaves from their best frequency. Because of the relatively small number of multiunits, we refrained from comparing effect sizes across the three brain regions.

Despite the relatively large number of error trials, their number was not sufficient to determine the phasic responses of individual multiunits in incorrect trials because there was no complete set of tone steps with at least three repetitions of each exemplar. To compare correct with error trials nonetheless, we constructed population responses from the multiunits in each of the three brain regions. For each multiunit, a list of trials was generated that allowed to create one or more sets of four randomly selected trials such that a set consisted of two tone steps which ended on the same frequency, which were of the same size (0.5 or 1 octave) but of opposite direction (downstep and upstep), and which yielded either a correct (‘hit’, ‘correct rejection’) or an incorrect (‘miss‘, ‘false alarm’) behavioral response. In each set, the phasic response increases were rank ordered, with a rank of 1 assigned to the smallest increase and a rank of 4 assigned to the greatest increase. Subsequently the four trials of the current set were removed from the list, and another set of four trials was formed accordingly. This selection was continued until no further complete sets with the four trial types could be formed. The procedure of set formation was performed on all multiunits, such that the average ranks for each of the four trial types could be computed from all sets of trials of all multiunits in each of the three brain regions. Because the sets of trials were randomly composed the result of this procedure varied with set composition. Thus we could repeat the procedure of computing the average rank for the four trial types 1000 times, allowing us to perform statistical tests across the average rank of the four trial types.

#### Tonic responses

To assess whether a multiunit exhibited a tonic response during a specific phase of the auditory categorization task we computed another PSTH with a bin size of 200 ms which was triggered on the onset of the tone sequence and which included all tone sequences, irrespective of the initial frequency. The firing rate was normalized by dividing it through the mean firing 200 ms before cue light onset. A multiunit was classified to exhibit a tonic response during a specific phase if its firing in a 200-ms pre-period before the phase was significantly different from the firing within the last 200 ms of the phase (Wilcoxon-test, p < 0.001). In this report three phases of the task were analyzed: (1) the *waiting period* (because of the presence of grasping-related firing [Brosch et al., 2005] we used the 200-ms interval immediately before cue light onset as the pre-period), (2) the *initial tones* of the sequence before categorization (from the onset of the tone sequence until the onset of the 4^**th**^ tone), and (3) the *intermittent tones* of the sequence after categorization (from the onset of the 4^**th**^ tone until the onset of the 8^**th**^ tone and only using sequences with upsteps). Since the control monkey did not perform a task, the pre-period of the waiting period started 2420 ms before sequence onset.

We performed a choice probability (CP) analysis to determine statistically whether the neuronal firing was correlated with the animals' choice in different time windows during a trial ([24]). Similar to Niwa and colleagues [21], we compared the distribution of firing rates in a 200-ms time window of interest in correctly performed trials with the corresponding distribution in incorrectly performed trials. For sequences with intermittent tones we compared false alarms with correct rejections. For sequences without intermittent tones we compared hits with misses. Then, using Receiver Operating Characteristic (ROC) curve analysis, i. e., we determined, for different criterion levels, the proportions of trials with and without bar releases in which the firing rate was greater than a given criterion level. The criterion level varied from the lowest to the highest firing rate in 100 equal steps. CP was defined as the area under the resulting ROC curve. For time windows after the 4^th^ tone, individual trials were analyzed only until bar release. This was done because after bar release the tone sequence was stopped such that trials with and without bar release also differed in the absence or presence of the tones. It also precluded that the CP analysis was affected by neuronal activity related to bar release ([25]). Therefore CPs of individual multiunits were based on fewer trials in these time windows than in earlier time windows. Because the tone sequence stopped at the latest at 2400 ms in trials with hits and at the latest at 2800 ms in trials with false alarms, CPs could be calculated for two more 200-ms time windows when false alarms were compared with correct rejections than when hits were compared with misses.

## Results

Results of the present report are based on multiunit activity recorded from 641 sites in the right auditory cortex (RAC), the right auditory thalamus (MGB) and the left auditory cortex (LAC) during 187 sessions in three monkeys. Table 1 summarizes, for each of the three brain regions and for each of the three monkeys, the total number of sites, the number of sites that were used for the analyses of phasic responses to tone steps, the number of sessions, and the average percentages of correct trials in these sessions.

**Table 1:** Overview of the sample sizes used in the current report.

		RAC	MGB	LAC
Total number of sites	**Monkey B**	183	32	134
	**Monkey F**	56	18	175
	**Monkey C**	**—**	—** **	43
Number of sites used for analyses of category responses	**Monkey B**	31	11	26
	**Monkey F**	3	9	10
	**Monkey C**	—	—	28
Number of sessions	**Monkey B**	51	17	40
	**Monkey F**	18	7	40
	**Monkey C**	—	—	14
Percentage of correct trials	**Monkey B**	71	69	71
	**Monkey F**	80	75	77
	**Monkey C**	—	—	—

The table shows the total number of sites, the number of sites that were used for the analyses of phasic responses to the three categories of tone steps, the number of training sessions, and the percentages of correct trials in these sessions separately for each of the three monkeys and for the right auditory cortex (RAC), the right auditory thalamus (MGB) and the left auditory cortex (LAC).

### Phasic responses to tone steps

As with LAC ([12]), we found category-specific neuronal responses in RAC and in MGB. The dot rastergrams in Fig 3 show when an example multiunit from RAC (panel A) and an example multiunit from MGB (panel B) fired spikes relative to the presentation of individual exemplars of downsteps (green dots), upsteps (red dots), and frequency repeats (blue dots). The downsteps and upsteps had a size of 0.5 octaves (pale dots) or 1 octave (bold dots) and all exemplars ended on trigger tones within the same 1.5-octave frequency range. Inspection of the dot density indicated that the responses to the trigger tone depended on their relationship to the best frequency and the frequency response area (see insets), the size of the tone steps ([31]) and, of largest interest in the current study, on the direction of the tone steps. When the responses to all exemplars of each of the three classes of tone steps were combined, both multiunits were found to exhibit a stronger phasic response to downsteps than to upsteps or to frequency repeats (bottom row in Fig 3).

**Fig 4:**
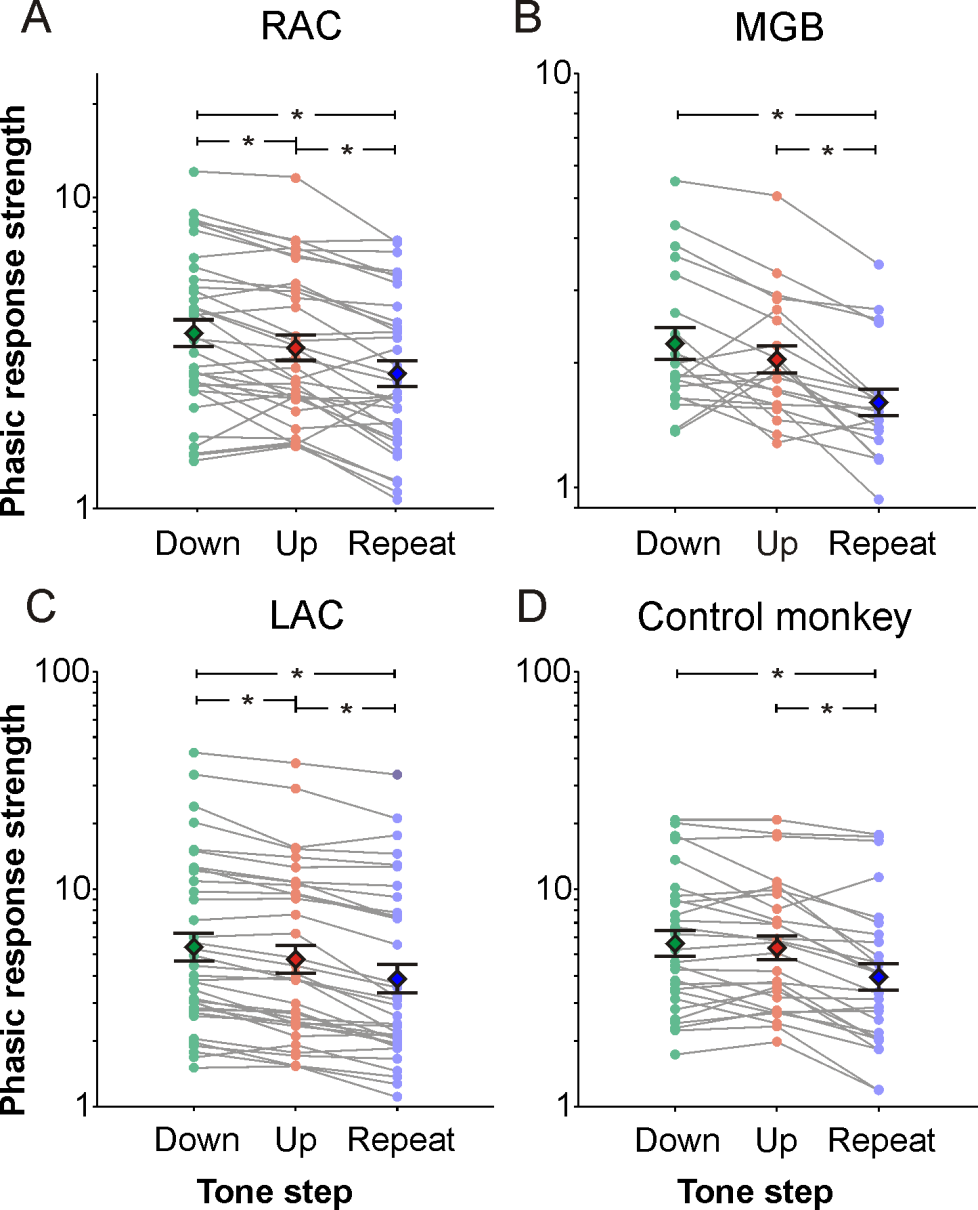
Similar preferences for downsteps in three regions of the auditory forebrain. (A) Distributions of phasic response strengths for downsteps, upsteps, and frequency repeats of 34 multiunits in right auditory cortex (RAC). Data from the same multiunit are connected by lines. The diamonds represent mean logarithmic strengths and the whiskers represent standard errors. Asterisks mark significant differences between pairs of tone steps (t-tests, p < 0.01). Phasic response strength was defined as the maximal normalized firing rate (see lower panels in Fig 3). (B) Distributions of phasic response strengths for the three tone steps of 20 multiunits in right medial geniculate body (MGB). (C) Distributions of phasic response strengths for the three tone steps of 36 multiunits in left auditory cortex (LAC). (D) Distributions of phasic response strength for the three tone steps of 28 multiunits in the LAC of a control monkey who was naive to the categorization task and only passively exposed to the tone sequences.

Generally, neurons in RAC and MGB responded more strongly to downsteps than to upsteps and to frequency repeats. This was found when we analyzed 34 tone-responsive multiunits in RAC and 20 of such multiunits in MGB (see Methods). In RAC, 23 of the 34 multiunits responded more strongly to downsteps than to upsteps, and 32 responded more strongly to upsteps than to frequency repeats. These relationships were also reflected in the average phasic response strength of these multiunits, which was significantly greater for downsteps than for upsteps or frequency repeats, and significantly greater for upsteps than for frequency repeats (t-tests, each p < 0.01; Fig 4A). In MGB, 14 of the 20 multiunits responded more strongly to downsteps than to upsteps, and 17 responded more strongly to upsteps than to frequency repeats. This difference was also present in the average phasic response strength of these multiunits (Fig 4B), which was significantly different between downsteps and frequency repeats as well as between upsteps and frequency repeats (p < 0.01), but which failed to reach significance for the comparison between downsteps and upsteps (p = 0.07). The same relationships between tone steps and phasic response strengths were seen in 36 multiunits in LAC (Fig 4C). These were selected from the 59 multiunits which we had previously analyzed ([12]), such that the best frequencies in the three brain regions were from a similar range (140 to 22880 Hz).

**Fig 5:**
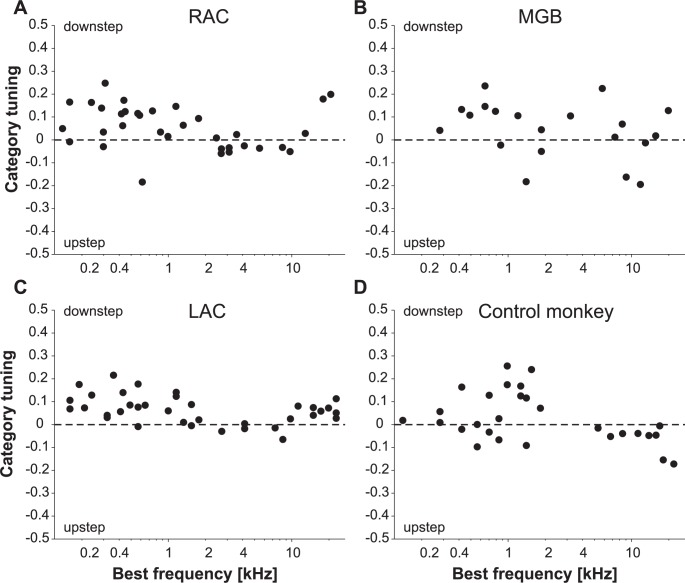
Category tuning is independent of best frequency in three regions of the auditory forebrain of trained monkeys. (A-C) Results for the 34 multiunits in RAC, the 20 multiunits in MGB, and the 36 multiunits in LAC. Category tuning was calculated by dividing the difference of the responses to downsteps and upsteps through the sum of the two responses. Positive values indicate preference for downsteps. (D) In the LAC of the control monkey, category tuning differed between multiunits with low and high best frequencies (below and above 3.5 kHz).

It is unlikely that the differences between the responses to downsteps and upsteps in the three brain regions resulted from sampling preferentially from multiunits whose best frequency better matched the trigger tone. This is supported by finding no systematic differences in the category tuning in the three brain regions when we tested with tones near the best frequency or with tones up to four octaves from the best frequency. Category tuning was defined as the difference of the responses to downsteps and upsteps divided by their sum. Further support comes from finding the response preference for downsteps for the full range of best frequencies obtained in RAC, MGB, and LAC (Fig 5A–5C). Specifically, there was no statistically significant difference in category tuning between multiunits with low (< 3.5 kHz) and high (≥ 3.5 kHz) best frequencies in the three brain regions (t-test, each p > 0.05). This finding also argues against the possibility that the differences between the responses to downsteps and upsteps resulted exclusively from testing multiunits with tone steps whose first frequency induced different amounts of forward suppression for upsteps and downsteps. If this was the cause, multiunits with high best frequencies would be expected to respond more strongly to upsteps. This is because for the tone steps used here, the first tone of the upsteps was more frequently more distant from the best frequency than the first tone of the downsteps, and thus produced less forward suppression. The extent of the influence of the testing frequency range was observed in the control monkey in which 28 multiunits were analyzed during passive stimulation with the same tone sequences (Fig 5D). In this experimental group, multiunits with low and high best frequencies differed significantly in their category tuning (t-test, p = 0.007).

The results from the control monkey also indicated that the differences between the responses to downsteps and upsteps in the three brain regions were likely due to the extensive experience the monkeys had gained in categorizing tone steps during the course of the experiments. In the LAC of this monkey there was no significant difference of phasic response strengths between downsteps and upsteps (p = 0.27; Fig 4D). It is unlikely that the lack of difference between upsteps and downsteps in this monkey resulted from the fact that the tests were performed without task engagement because in the two trained monkeys, a preference for downsteps was also present during passive stimulation (see S4 Fig in [12]).

**Fig 6:**
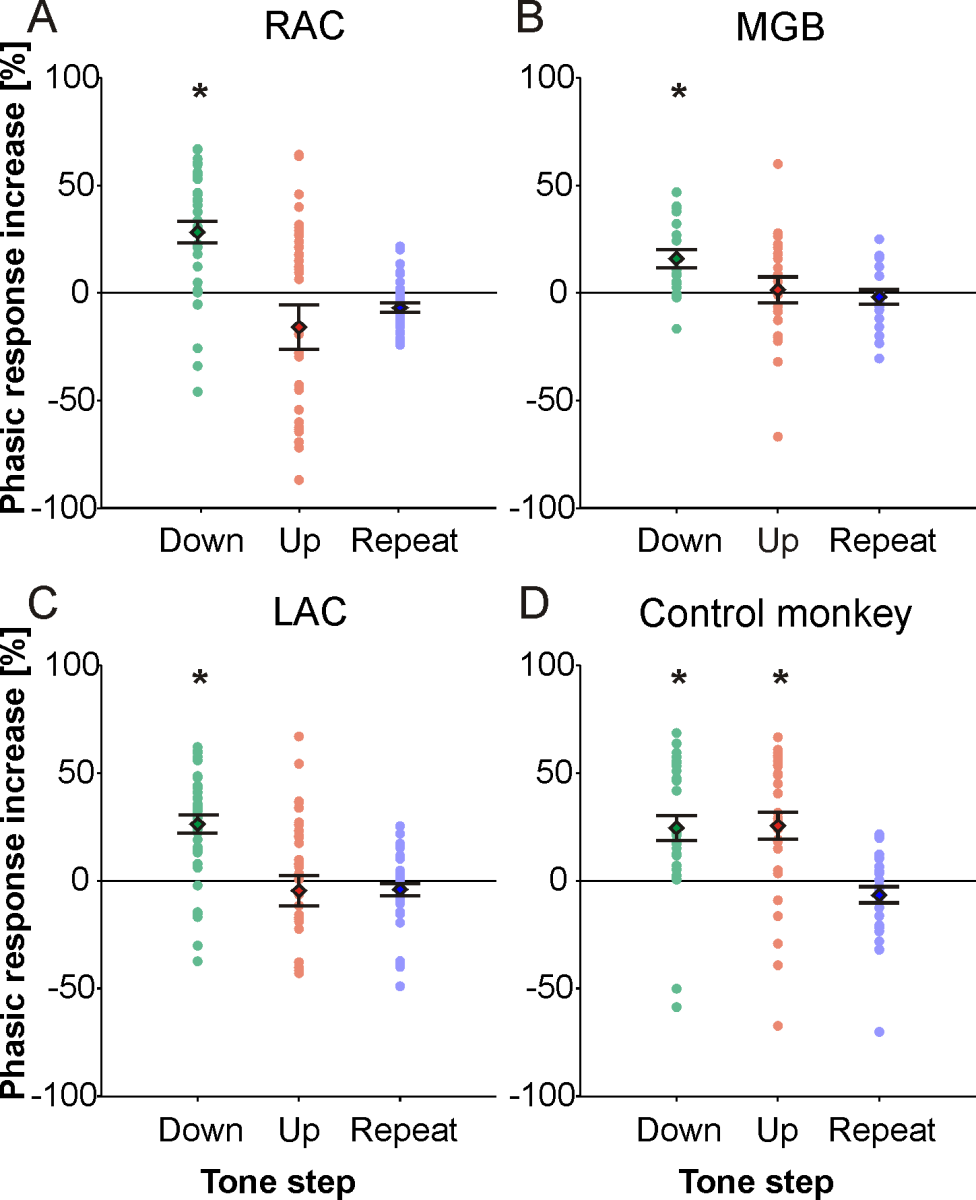
Distributions of phasic response increases in three regions of the auditory forebrain. Phasic response increases describe the temporal development of phasic response strengths within a trial (see Methods). Asterisks mark average phasic response increases significantly greater than 0 (t-tests, p < 0.01). All other conventions as in Fig 4.

The behavioral relevance of the three classes of tone steps, i. e., that both upsteps and tone repeats required bar holding while downsteps required bar release, could be better derived from the neuronal firing when we searched for the tone in the sequence that yielded a phasic response that was greater than the response to the preceding tone. This was quantitatively assessed by computing the phasic response increase for the three classes of tone steps (see Methods) and testing the hypothesis that the distribution of phasic response increases had a mean less than or equal to zero (t-test, p < 0.01). Analysis of the 34 multiunits in RAC (Fig 6A) and the 20 multiunits in MGB (Fig 6B) revealed that, on the population level, responses significantly increased only for downsteps but neither for upsteps nor for frequency repeats. This was also the case for our subsample of 36 multiunits in LAC (Fig 6C). Average phasic response increases varied between 16% and 28% for downsteps. A different picture emerged in the LAC of the control monkey (Fig 6D), where responses were significantly increased both for downsteps and upsteps but not for tone repeats. This observation supports the interpretation that the response increase exclusively for downsteps is due to learning-related neuronal plasticity.

**Fig 7:**
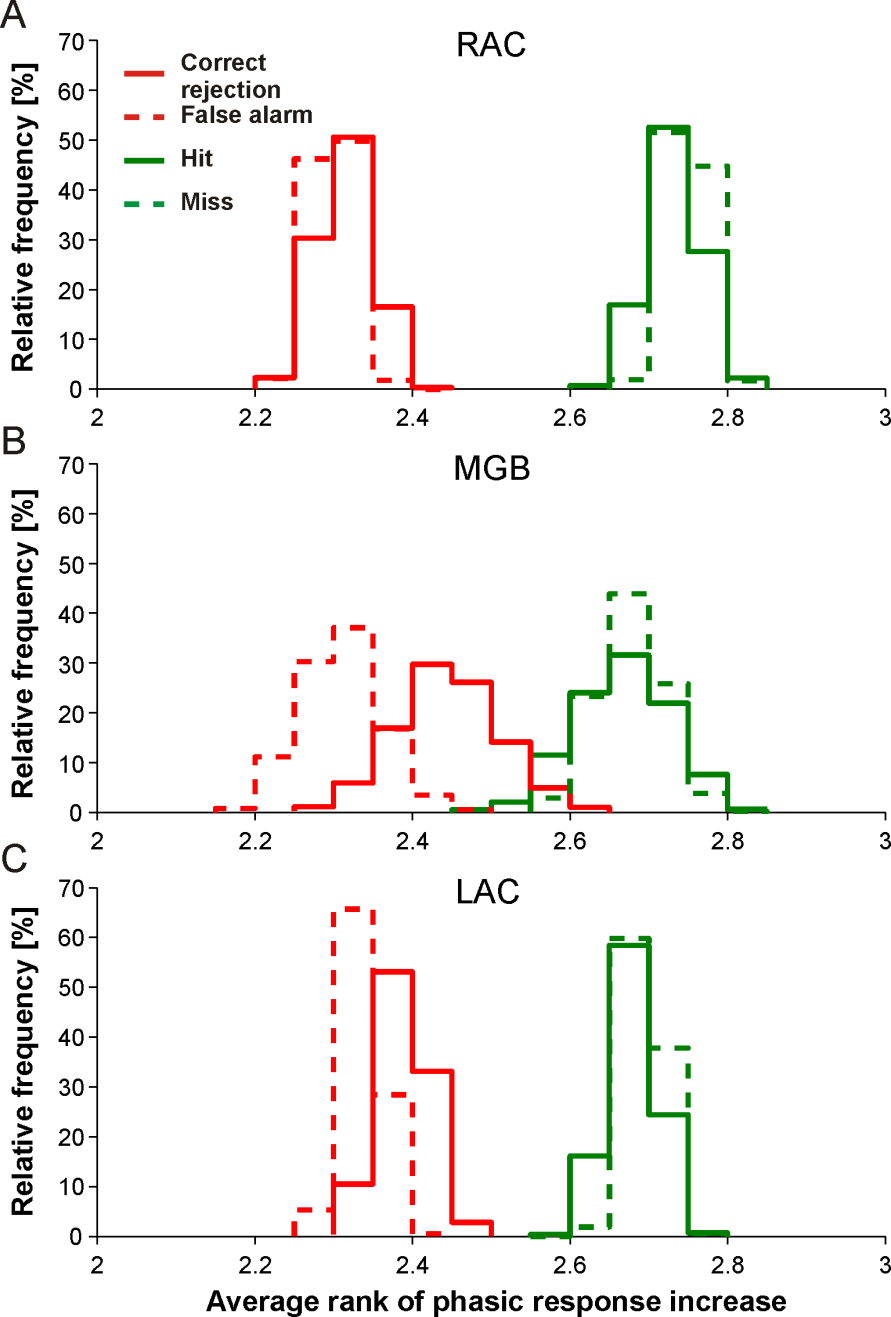
Similar discrimination of tone steps in correct and error trials in three regions of the auditory forebrain of trained monkeys. (A) The four histograms show the distributions of average ranks of phasic response increases calculated from 34 multiunits in RAC for upsteps (red curves) and downsteps (green curves), in trials with correct (solid lines) and incorrect behavioral responses (dashed lines). For each multiunit, the phasic response increases were rank ordered, with a rank of 1 assigned to the smallest increase and a rank of 4 assigned to the greatest increase. For further details see Methods. (B) Results for 20 multiunits in MGB. (C) Results for 36 multiunits in LAC.

We also found that the phasic response increases in the three brain regions were independent of how well the trained monkeys performed the tasks. This was obtained by computing the distributions of the average rank of phasic response increases for correctly and incorrectly recognized upsteps and downsteps in RAC (1760 trials), in MGB (520 trials), and in LAC (2244 trials; see Methods). Pairwise comparisons of the distributions revealed that RAC, MGB, and LAC discriminated downsteps from upsteps in correctly performed trials (hits and correct rejections) as well as in error trials (misses and false alarms; Fig 7).

### Tonic responses

**Fig 8:**
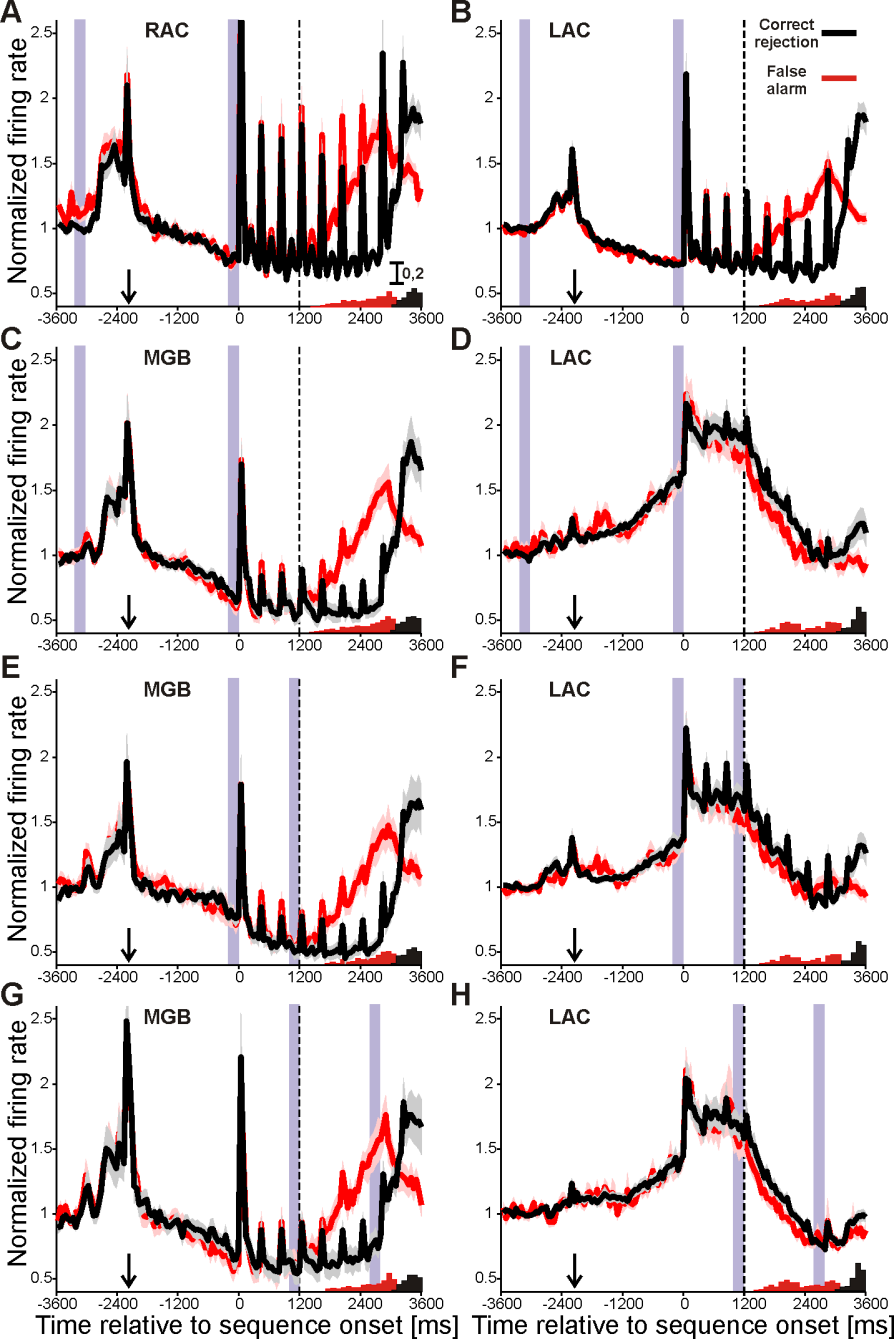
Tonic response types in three regions of the auditory forebrain of trained monkeys. (A) Time course of the normalized firing rate of 82 multiunits in RAC whose firing rate slowly decreased during the waiting period. The two purple vertical bars mark the time windows that were used to select these multiunits. The vertical dashed line marks the onset of the 4th tone. The black curve was calculated from trials in which the monkey correctly responded to the downstep after the presentation of the intermittent tones. The relative frequency of response times is shown in the lower right histogram (black bars). The red curve was calculated from trials in which the monkeys incorrectly responded to the upstep. The relative frequency of incorrect response times is shown in the lower right histogram (red bars). The shaded curves reflect the standard errors of the mean for each of the two curves. The increased firing about 2000 to 2800 ms before sequence onset is related to bar grasping and to the onset of the cue light. Firing before bar grasping (marked by the arrow) is dispersed because bar grasps occurred after variable reaction times after the cue-light. (B) Results of 127 multiunits in LAC whose firing rate decreased during the waiting period. (C) Results of 26 multiunits in MGB whose firing rate decreased during the waiting period. (D) Results of 61 multiunits in LAC whose firing rate increased during the waiting period. (E) Results of 19 multiunits in MGB whose firing rate decreased during the initial tones of the sequence. (F) Results of 72 multiunits in LAC whose firing rate increased during the initial tones of the sequence. (G) Results of 11 multiunits in MGB whose firing rate increased during the intermittent tones of the sequence. (H) Results of 89 multiunits in LAC whose firing rate decreased during the intermittent tones of the sequence.

Similar to LAC ([12], [13]), neurons in RAC and MGB also exhibited tonic responses during specific phases of the auditory task (Table 2). Tonic responses were already observed before the tone sequence started, i. e., after presentation of the cue light when the monkeys were holding the bar and waiting for sequence onset. During this waiting period, 34% of the multiunits in RAC (82/239), and 52% of the multiunits in MGB (26/50), slowly decreased their firing. The percentage of such multiunits was comparable to that in LAC, where 41% of them slowly decreased their firing (127/309). The average time courses of the slow firing decreases are shown in Fig 8A–8C. In LAC, there was also a sizeable percentage of multiunits (20%, 61/309) that slowly increased their firing during the waiting period (Fig 8D). The percentage of such multiunits in RAC and MGB was small (≤5%) and similar to the percentage in the LAC of the passively stimulated control monkey (chi-square tests, χ = 0.6 in RAC and χ = 0.01 in MGB).

**Table 2:** Percentage of multiunits with tonic responses in different brain regions of trained monkeys and of a control monkey.

	Trained			Control
	RAC	MGB	LAC	LAC
Waiting period (decrease)	34	52	41	0
Waiting period (increase)	5	2	20	2
Initial tones (decrease)	13	38	6	0
Initial tones (increase)	10	6	23	9
Intermittent tones (decrease)	4	2	29	0
Intermittent tones (increase)	3	22	2	0

The 'Waiting period' was the 2220-ms period before sequence onset. The 'Initial tones' comprised the first three tones of the sequence. The 'Intermittent tones' comprised sequences with upsteps and lasted from the 4th tone to the 8th tone. During these periods the multiunit activity either slowly decreased or increased.

Many multiunits also exhibited a tonic response during the presentation of the tone sequence which was superimposed on the phasic responses to the individual tones. During the three initial tones of the sequence, the three brain regions differed in the type of tonic response that prevailed. In MGB, multiunits preferentially decreased their firing (Fig 8E). In LAC, multiunits preferentially increased their firing (Fig 8F). For the remaining types of tonic responses in RAC, MGB and LAC, the percentages of multiunits were small and comparable to those in LAC of the passively stimulated control monkey.

During the following intermittent tones of the sequence (when four tones of higher frequency were presented), larger percentages of multiunits with tonic responses were found in MGB and LAC only, and their predominant type of tonic response was opposite to that during the initial tones. Thus multiunits in MGB preferentially increased their firing (Fig 8G, black curve) and multiunits in LAC preferentially decreased their firing (Fig 8H, black curve).

A strong association between the different types of tonic responses was only observed in LAC. Here, in many of the multiunits the firing increased during the waiting period, increased further during the initial tones and then decreased during the intermittent tones. They comprised 40 of the 309 multiunits in LAC (12.9%). The average time course of their firing was previously shown (Fig 4A in [12]).

In contrast to the phasic response to the 4^th^ tone, after which the monkeys differentiated upsteps from downsteps, the subsequent tonic firing in all three brain regions was partially related to how the monkeys responded in a trial. This can be gleaned from the population Post-Stimulus Time Histograms (PSTHs) shown in Fig 8. In many neurons the firing shortly after the 4^th^ tone (vertical dashed line) determined whether the monkeys would or would not release the touch bar to the tone step. In the neuronal populations from LAC, RAC and MGB shown in panels A-C, D, and E, the firing was higher when the monkeys (incorrectly) released the bar to an upstep (red curves) compared to when the monkeys (correctly) refrained from releasing the bar to an upstep (black curves). Similarly, we found that the firing of the same neurons was higher when the monkeys (correctly) released the bar to a downstep compared to when the monkeys (incorrectly) missed releasing the bar to a downstep (not shown). The main reason for the difference was that many neurons exhibited firing that was related to bar release, as previously described for LAC ([25]). In addition, there were neuronal populations in LAC with lower firing rates when the monkeys released the bar than when they did not release the bar (panels D, F, and H).

**Fig 9:**
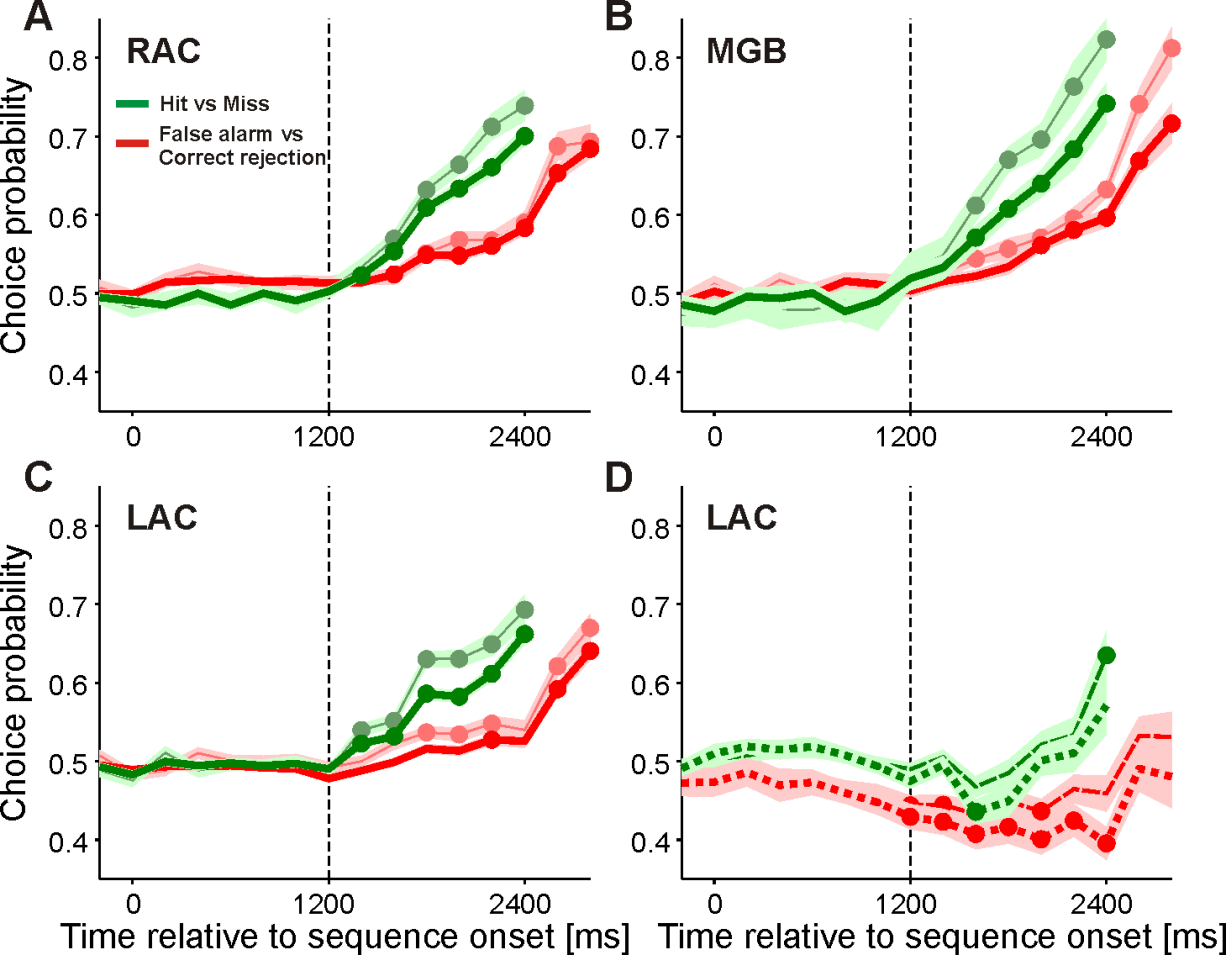
Time courses of choice probabilities after sequence onset in three regions of the auditory forebrain. (A) The thick lines show mean CPs of 239 multiunits recorded in RAC. The thin lines show mean CPs of 82 multiunits recorded in RAC whose firing rate slowly decreased during the waiting period (cp. Fig 8A). The two green curves represent comparisons between hits and misses. The two red curves represent comparisons between false alarms and correct rejections. The shaded curves reflect the standard errors of the mean for each of the four curves. Note that for the latter comparisons, CPs of two more 200-ms time windows could be shown. The dots represent CPs significantly different from 0.5 (t-test, p < 0.001). The vertical dashed line marks the 4th tone. (B) CPs of all 50 multiunits recorded in MGB (thick lines) and of 26 multiunits in MGB whose firing rate slowly decreased during the waiting period (thin lines; cp. Fig 8C). (C) CPs of all 309 multiunits recorded in LAC (thick lines) and of 127 multiunits in LAC whose firing rate slowly decreased during the waiting period (thin lines; cp. Fig 8B). (D) CPs of 61 multiunits in LAC whose firing rate slowly increased during the waiting period (dashed curves; cp. Fig 8B) and of 40 multiunits in LAC whose firing rate increased during the waiting period, also increased during the initial tones and then decreased during the intermittent tones (dotted curves).

We performed a choice probability (CP) analysis to quantitatively assess these relationships between the neuronal firing and the behavioral responses of the monkeys to allow better comparison with previous studies on decision related activity ([18], [19], [22]). This analysis confirmed that shortly after the 4^th^ tone, neuronal firing in the three brain regions reflected the monkeys' choices. The mean CPs computed from all 239 multiunits in RAC, 50 multiunits in MGB, and 309 multiunits in LAC were indistinguishable from chance (CP = 0.5) during the waiting period and the initial tones of the sequence, and became significantly greater than 0.5 after the 4^th^ tone (t-tests, each p < 0.001). This was observed both when we compared false alarms with correct rejections (thick red curves in Fig 9A–9C) and hits with misses (thick green curves). CPs increased to maximal values at different times after the 4^th^ tone for the two comparisons, possibly reflecting that the monkeys released the touch bar 415 ± 128 ms earlier in trials with correct responses to downsteps (hits) than in trials with incorrect responses to upsteps (false alarms). These relationships between the neuronal firing and the behavioral responses of the monkeys were observed in many multiunits in the three brain regions, including those of the five subpopulations with the previously described tonic response types (Fig 8A–8C, 8E and 8G). This is shown by the thin lines in Fig 9A–9C for the subpopulations of multiunits in RAC, MGB, and LAC that slowly decreased their firing during the waiting period. A different relationship between neuronal firing and behavioral responses was observed for some of the other subpopulations identified in LAC. Their mean CPs were either not significantly different from 0.5 or even smaller than 0.5 after the 4^th^ tone. These subpopulations consisted of multiunits that increased their firing during the waiting period (dashed lines in Fig 9D), increased their firing during the initial tones, or decreased their firing during the intermittent tones (both not shown). The largest effects were observed in the CPs of the 40 multiunits which combined these three tonic response types (dotted lines in Fig 9D). Neurons with CPs above or below 0.5 may be differentially involved in the categorization task utilized here. Neurons with CPs greater than 0.5 may eventually reach a critical firing rate sufficient to contribute to bar release. They frequently exhibited firing that was related to bar release. Neurons with CPs less than 0.5 may promote continued bar holding. This is suggested by finding that these neurons frequently did not exhibit firing that was related to bar release.

## Discussion

This study indicated the presence of similar neuronal mechanisms in early auditory cortical areas of the left (LAC) and the right hemisphere (RAC) for the categorization of tone intervals. In both hemispheres, the task of reporting a downstep by releasing a bar involved neurons that exhibited phasic responses with short latencies and durations whose magnitude differed between downsteps, upsteps and frequency repeats. These response differences were found both in trials with correct and incorrect reports, indicating that these neuronal responses at least partially contributed to the monkeys' behavioral choices. A stronger relationship between neuronal firing and choice behavior, however, was sometimes seen after the presentation of tone steps, when many of the neurons in the two hemispheres exhibited higher firing rates in trials with bar releases than in trials with no bar releases. These neurons were supplemented by other neurons we only observed in LAC but not in corresponding regions of RAC, which had the opposite relationship between the neuronal firing and the monkeys' choices. These neurons differed from the remaining neurons in LAC and RAC in that they displayed a specific type of tonic response before the tone sequence, i. e., their firing rate slowly ramped up. Our observation that choice-related activity was not yet present at the moment a meaningful auditory stimulus was presented but emerged some time later is consistent with the observations of some other studies on choice-related activity in the auditory cortex. In a study in which monkeys categorized human speech sounds, auditory responses to such sounds in the lateral belt areas also did not reflect the monkeys' choices ([18]). In a subsequent study [19]) in which monkeys categorized whether an auditory stimulus contained more low-frequency or high-frequency tone bursts choice-related activity emerged in the lateral belt around the time of decision. By contrast, in a study in which monkeys were cued by a noise burst to discriminate an amplitude modulated sound from an unmodulated sound, choice-related activity emerged both in lateral belt and primary auditory cortex already before the discrimination ([21], [22]). Choice activity was also observed in different auditory cortical fields of ferrets performing a pitch judgement task ([23]) but not in primary auditory cortex of monkeys performing a flutter discrimination task ([20]). The differences between the studies may reside in differences in task demands (categorization vs discrimination) and corresponding reaction times.

Our observations of largely similar neuronal responses to tone steps in LAC and RAC is consistent with results of previous studies in early auditory cortical areas of the left and the right hemisphere. No differences were found in the functional organization of response properties in left and right primary auditory cortex of marmosets ([32]) and in the responses to click trains in primary and posterior auditory cortex of macaques ([33]). Likewise, similar BOLD activations were found in the left and right primary auditory cortex of monkeys when monkey and human vocalizations were compared to scrambled versions of them ([34]). Thus, it is possible that different roles of LAC and RAC for the discrimination of the direction of tone steps ([14]) and frequency sweeps ([16], [17]) and for other aspects of audition ([35], [36], [37], [38], [39],[40]) do not result from different phasic responses or auditory receptive fields but are based on differences of tonic responses. Alternatively, the different roles of LAC and RAC might be based on differences that cannot be observed in the neuronal firing, or on differences reflected in auditory cortical areas other than those studied here. It is also possible that such differences are not present in nonhuman primates.

In the auditory thalamus, the neuronal activity associated with the categorization of pitch relationships was found to be largely similar to that in primary and caudomedial auditory cortex. Phasic responses discriminated tone steps, tonic responses were related to specific phases of the categorization task, and some neurons could displayed choice-related firing. Although we could not exactly reconstruct the location of the recording sites in the auditory thalamus it is likely that many of them were located in the ventral division of the right MGB. This suggests that primary auditory cortex receives category-specific input from the ascending auditory system. Whether this input fully determines the selectivity of phasic responses in auditory cortex, or whether additional modifications are taking place, was not addressed in the current experiments. The sources of tonic responses and choice-related activity in MGB and auditory cortex are unknown, but may include the prefrontal cortex ([41]) or basal ganglia ([42]).

The current study corroborates and extends the knowledge of roles of MGB in auditory cognition ([43]). Here we provide further evidence for category-related neuronal responses in the MGB ([44]). Earlier studies reported that about two thirds of thalamic neurons respond either more strongly, or more weakly, to auditory stimuli when monkeys were engaged in an auditory task than when they are only passively stimulated ([45], [46], [47]), or when rats had to change the association between sounds and actions ([48]). In patients, electrical stimulation of left but not of right ventrolateral thalamus improved the persistence of verbal material in short-term memory, while the same intervention deteriorated performance during recall ([49]). Involvement of MGB in cognitive and motivational circuits is also suggested by our findings of tonic responses in this part of the auditory system, i. e., of a signal that has been suggested to reflect associations between elements of tasks ([13], [50]).

To the best of our knowledge, our study provides the first experimental support for the presence of choice-related neuronal firing in the sensory thalamus. Previous studies on rodents and nonhuman primates did not report such activity in the auditory ([44]) and in the somatosensory thalamus ([51], [52]). Very recently some weak and transient choice-related activity was observed in the ventral posteromedial nucleus of the thalamus ([53]). This activity, however, could not account for the prolonged choice-related activity in the somatosensory 'whisker' cortex. A possible explanation for the lack of choice-related activity in auditory and somatosensory thalamus is that only early sensory-related activity was analyzed in the cited studies. Indeed, closer inspection of Fig 2 in Vazquez et al. [51] suggests the presence of movement-related activity in somatosensory thalamus towards the end of the detection task. Despite this, increases of CP above 0.5 were not reported because for the CP analysis the authors compared trials with pressing of the correct response key to trials with pressing of the incorrect response key. This differs from the present study in which trials with bar releases were compared to trials without bar releases.

We obtained no evidence for different category-specific phasic responses to upsteps and downsteps in the auditory cortex of a control monkey who was naive to the task of categorizing tone steps. This suggests that the extensive training the two monkeys used in the current study had received on this task had induced long-lasting changes of the neuronal responses to tone steps in the auditory forebrain. Our study thus demonstrates that learning not only causes changes in neuronal response properties for specific frequencies or sound intensities (reviewed in [54]) but can also cause changes in response properties for entire stimulus classes or categories. It is possible that the extensive training resulted in a selective decrease of the phasic response to upsteps, thereby increasing the contrast between the responses to the different tone steps. We expect that the use of other task contingencies, e. g., associating the three classes of tone steps with three behavioral responses in a different way, would create three separate neuronal populations in the auditory forebrain. Indication for different neuronal representations has recently been obtained in the primary auditory cortex of animals that were also trained to categorize tone steps ([55]).

In conclusion, the current study provides additional support for roles of auditory core and belt fields beyond stimulus processing. We confirm the existence of phasic neuronal activity related to auditory categories ([12], [18], [44], [56], [57]), of tonic activity related to anticipating, memorizing, and associating stimuli ([13]), of activity related to motivational task aspects, including reward ([28]), of activity related to attention ([58]) as well as of activity related to choice ([19], [21], [22], [23]).
